# XIAP Interaction with E2F1 and Sp1 via its BIR2 and BIR3 domains specific activated MMP2 to promote bladder cancer invasion

**DOI:** 10.1038/s41389-019-0181-8

**Published:** 2019-12-06

**Authors:** Jiheng Xu, Xiaohui Hua, Rui Yang, Honglei Jin, Jingxia Li, Junlan Zhu, Zhongxian Tian, Maowen Huang, Guosong Jiang, Haishan Huang, Chuanshu Huang

**Affiliations:** 1 Nelson Institute of Environmental Medicine, New York University School of Medicine, 341 East 25th Street, New York, NY 10010 USA; 2 Zhejiang Provincial Key Laboratory for Technology and Application of Model Organisms, Key Laboratory of Laboratory Medicine, Ministry of Education, Wenzhou, China; 3 School of Laboratory Medicine and Life Sciences, Wenzhou Medical University, Wenzhou, Zhejiang China

**Keywords:** Bladder cancer, RHO signalling

## Abstract

XIAP has generally been thought to function in bladder cancer. However, the potential function of structure-based function of XIAP in human BC invasion has not been well explored before. We show here that ectopic expression of the BIR domains of XIAP specifically resulted in MMP2 activation and cell invasion in XIAP-deleted BC cells, while Src was further defined as an XIAP downstream negative regulator for MMP2 activation and BC cell invasion. The inhibition of Src expression by the BIR domains was caused by attenuation of Src protein translation upon miR-203 upregulation; which was resulted from direct interaction of BIR2 and BIR3 with E2F1 and Sp1, respectively. The interaction of BIR2/BIR3 with E2F1/Sp1 unexpectedly occurred, which could be blocked by serum-induced XIAP translocation. Taken together, our studies, for the first time revealed that: (1) BIR2 and BIR3 domains of XIAP play their role in cancer cell invasion without affecting cell migration by specific activation of MMP2 in human BC cells; (2) by BIR2 interacting with E2F1 and BIR3 interacting with Sp1, XIAP initiates E2F1/Sp1 positive feedback loop-dependent transcription of miR-203, which in turn inhibits Src protein translation, further leading to MMP2-cleaved activation; (3) XIAP interaction with E2F1 and Sp1 is observed in the nucleus. Our findings provide novel insights into understanding the specific function of BIR2 and BIR3 of XIAP in BC invasion, which will be highly significant for the design/synthesis of new BIR2/BIR3-based compounds for invasive BC treatment.

## Introduction

The X-linked inhibitor of apoptosis (XIAP) is an IAP protein family member and a well-defined inhibitor of the caspase/apoptosis pathway^1–3^. XIAP overexpression is particularly associated with the progression and aggression of malignant cancer^4^. There are four functional domains in XIAP: three repeats of the baculovirus IAP repeat (BIR) domain at its NH_2_ terminus and a RING finger domain near its COOH terminus. The BIR domains are mainly responsible for XIAP’s anti-apoptotic function by inhibiting caspase-3, -7 and -9. The RING domain contains E3 ubiquitin ligase activity, which allows IAPs to ubiquitinate itself, caspase-3, and caspase-7^5^. The studies from our laboratory and others reveal novel functions of XIAP beyond the anti-apoptotic function^6,7^. For example, XIAP upregulates cyclin D1 expression *via* an E3 ligase-mediated protein phosphatase 2A/c-Jun axis^8^ and upregulates cyclin E expression as a result of the direct binding of E2F1 by the BIR domains, which promotes human colon cancer cell growth^9^. XIAP also enhances human invasive BC cell proliferation due to the BIR domain-mediated *c-Jun/miR-200a/EGFR* axis^10^. The RING domain of XIAP interacts with RhoGDIα protein to inhibit RhoGDIα SUMOylation at Lys-138, subsequently affecting human colon cancer cell migration^11,12^. Moreover, downregulation of the tumor suppressor p63α protein expression by the RING domain of XIAP promotes malignant transformation of bladder epithelial cells^13^.

Matrix metalloproteinases-2 (MMP2) belongs to the family of MMPs that can degrade the connective tissue stroma and basement membranes^14^. In mammalian cells, MMP2 mainly exists in two forms: pro-MMP2 and activated MMP2. Pro-MMP2 turns into activated MMP2 *via* proteolytic cleavage or chemical disruption to remove its pro-domain^15^. It has been reported that high expression of MMP2 could promote BC cell metastasis^16^. Our previous findings also showed that MMP2 is increased in BBN-induced mouse BC tissues and plays a critical role in BC cell metastasis^17,18^. However, MMP2 activation in BCs remains little known.

Our current studies emphasized the novel role of specific BIR2 and BIR3 domains of XIAP on BC cancer invasion and reveal that XIAP promoted BC invasion through its BIR domains, indicating a previously underappreciated role of BIR2/3 domains in the promotion of the invasive activity of BC cells. Thus, we further examined the signaling pathways and functional XIAP cellular localization that relate to this important function in the current study. We have discovered that this novel function is mediated by the specific activation of MMP2 due to BIR domain-initiated suppression of Src protein translation. Moreover, the BIR domains of XIAP attenuated Src protein translation due to interaction of BIR2 and E2F1 as well as BIR3 and Sp1, leading to miR-203 transcription and it’s binding to *Src* mRNA 3′-UTR region.

## Materials and methods

### Cell lines, plasmids, antibodies, and other reagents

The human invasive BC cell line UMUC3 was provided by Dr. Xue-Ru Wu (Department of Urology and Pathology, New York University School of Medicine, New York, NY), and was used in our previous studies^17,19^. The human metastatic BC cell line T24T, which is a lineage-related metastatic lung variant of the invasive BC cell line T24^20^, was kindly provided by Dr. Dan Theodorescu^21^ and was used in our previous studies^22,23^. For the details of reagents, cell lines and cell culture, see the Supplement of “Materials and Methods”.

### Human bladder cancer tissue samples

Twenty pairs of primary bladder cancer samples and their paired adjacent normal bladder tissues were obtained from patients who underwent radical cystectomy at the Department of Urology at the Union Hospital of Tongji Medical College (Wuhan, China) between 2012 and 2013. For more details, please see the Supplement of “Materials and Methods”.

### Animal experiments and immunohistochemistry-paraffin (IHC-P)

Animal experiments and immunohistochemistry-paraffin were conducted according to protocol as we described previously^17^.

### Western blot

Western blots were assessed, as previously described^24^.

### RT-PCR and quantitative RT-PCR

Total RNA was extracted with TRIzol reagent (Invitrogen Corporation, CA, USA), and cDNAs were synthesized with a SuperScript III First-Strand Synthesis System for RT-PCR (Invitrogen Corporation, CA, USA). For more details, please see the Supplement of “Materials and Methods”.

### [^35^S] Methionine pulse new protein synthesis assays

[35S]-methionine/cysteine was employed to determine SRC protein translation as described in the Supplement of “Materials and Methods”.

### Statistical methods

Associations between the categorical variables were assessed using a chi-square test. Student’s *t*-test was utilized to compare continuous variables, and the results are summarized as the mean ± SD between different groups. Paired *t*-tests were performed to compare the difference between paired tissues in the real-time PCR analysis. *p* < 0.05 was considered statistically significant.

## Results

### XIAP BIR domains specifically promoted MMP2 activation and BC invasion in human BC cells

**Fig. 1:**
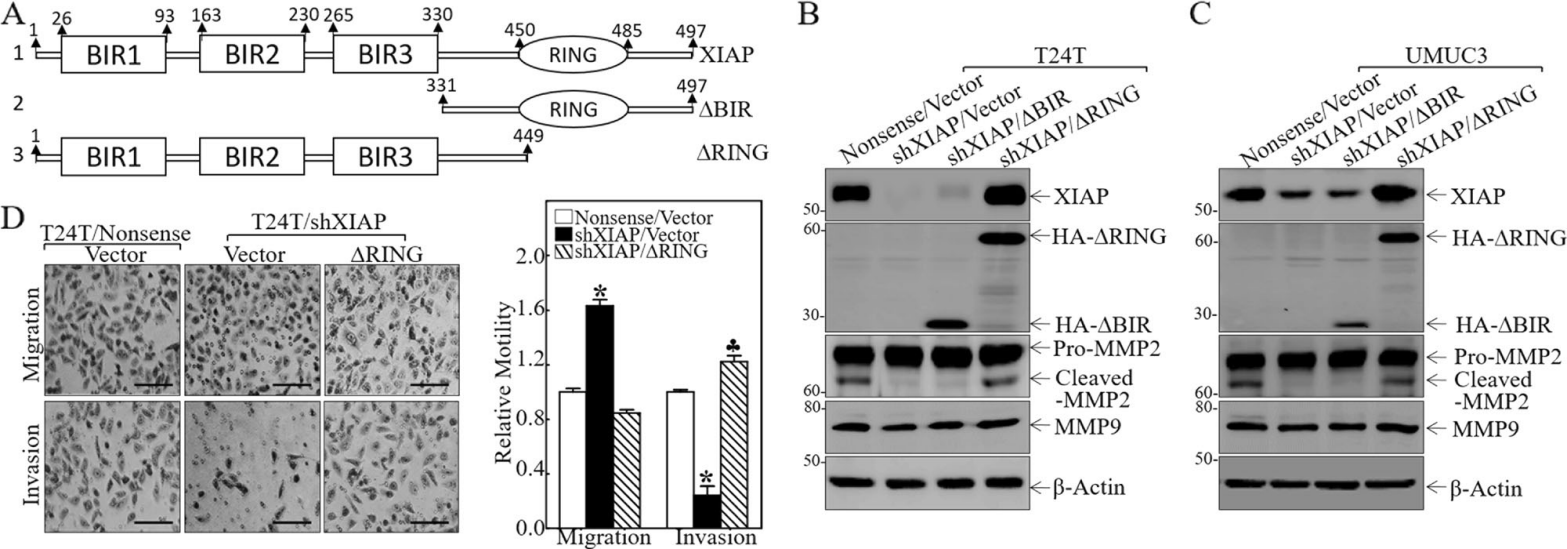
XIAP BIR domains promoted MMP2 activation and BC invasion. **a** The schematic structure of XIAP domains. **b**, **c** The indicated cell extracts were subjected to Western Blot to determine the expression of XIAP, MMP9, pro-MMP2, and cleaved-MMP2 (activated-MMP2). β-Actin was used as the protein loading control. **d** T24T (Nonsense/Vector), T24T (shXIAP/Vector), and T24T (shXIAP/ΔRING) cells were cultured in uncoated chambers or pre-coated Matrigel chambers for 24 h. The cells were then fixed and stained. Original magnification, ×100. Scale bars, 10 µm. The invasion and migration rates were quantified by counting the relative migratory (Transwell) and invasive cells in five random fields (*n* = 5) under a light microscope, then the cell numbers were normalized with the insert control according to the manufacturer’s instructions. The error bars show the mean ± SD of three independent experiments. The symbol (*) indicates a significant difference as compared with the vehicle control (*p* < 0.05) and the symbol (♣) indicates a significant difference compared with T24T(shXIAP/Vector) cells (*p* < 0.05).

XIAP contains three repeat BIR domains in the N terminus and one RING domain in the C terminus as schematically shown in Fig. 1a. We found that the reduction of MMP2 activation without affecting levels of pro-MMP2 protein and MMP9 protein by knockdown of XIAP expression was further reversed by ectopic expression of HA-ΔRING (XIAP in the presence of all three BIR domains), but not reversed by ectopic expression of HA-ΔBIR (XIAP in the presence of the RING domain) in T24T cells and UMUC3 cells (Fig. 1b, c). MMP2 degrades cellular matrix components and the basement membrane, and therefore reduces the barriers for cancer cell migration and/or invasion^25^. Our result showed that inhibition of XIAP expression dramatically reduces BC cell invasion (Fig. 1d). The reduction of BC cell invasion was restored when ectopic expression of ΔRING (Fig. 1d), indicating that BIR domains are crucial for XIAP-mediated BC invasion. Further, inhibition of XIAP expression increased cell migration (Fig. 1d), suggesting that although cancer cell invasion and migration are appealingly linked in many experimental systems, they may be divergent in their significance to and mechanism in human BC cells, as shown in our recently studies^26^.

### Src tyrosine kinase protein expression was inhibited by the XIAP BIR domains in BC cells and was also downregulated in human and mouse BC tissues

**Fig. 2:**
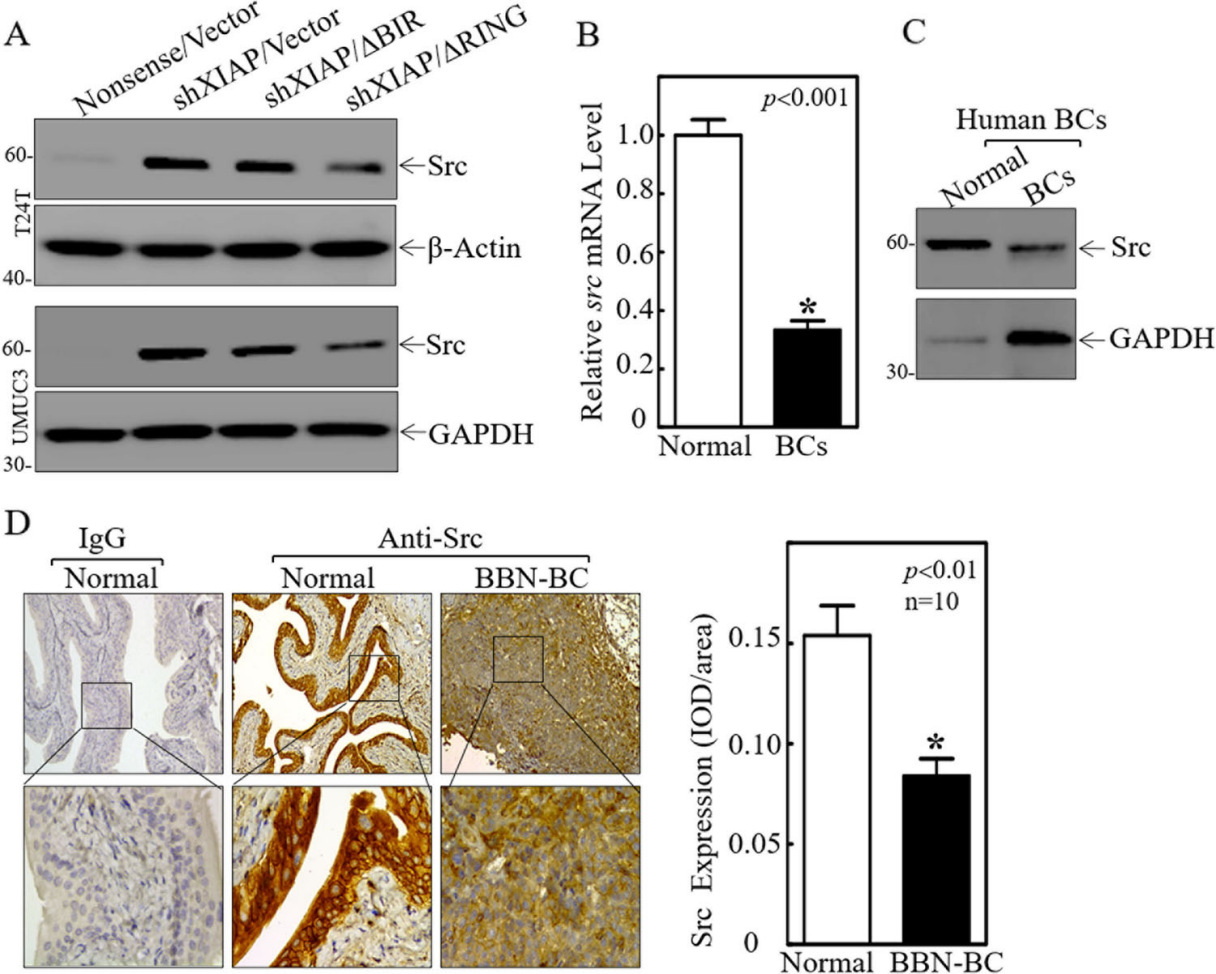
Src downregulated by XIAP BIR domains decreased in human BCs and mouse BCs. **a** The indicated cell extracts were subjected to Western blot to determine Src expression. **b**, **c** Total RNA and protein lysates were prepared from the mixture of equal aliquots of tumor tissue or the adjacent non-tumorous tissue from each of 20 BC patients and subjected to qPCR and Western blotting analyses to determine *Src* mRNA (**b**) and protein (**c**) expression profiles, respectively. **d** IHC-P was carried out to evaluate Src protein expression in mouse BC induced through consistent exposure of mice to BBN for 20 weeks. Original magnification, up: ×100, down: ×400. The optical density was analyzed as described in “Materials and methods”. The symbol (*) indicates a significant decrease in comparison to normal mice (*p* < 0.01).

It has been reported that decreased Src protein expression is associated with late-stage bladder tumor progression^27^. Interestingly, we found that attenuation of XIAP expression resulted in a profound increase in Src protein expression and this augmentation of Src protein expression was reversed by ectopic expression of HA-ΔRING, but not by HA-ΔBIR (Fig. 2a). Then we found that a profound reduction in *Src* mRNA expression was observed in human BC tissues, with an overall average of a threefold lower relative *Src* mRNA level in comparison to the normal controls (Fig. 2b). Consistent with the mRNA expression results, significantly decreased Src protein expression was also observed in human invasive bladder cancer tissues (Fig. 2c). Moreover, The results from the immunohistochemistry (IHC) staining reveal that Src expression was markedly decreased in mouse invasive BC tissues in comparison to normal mouse bladder tissues (Fig. 2d). In vitro, knockdown of Src in T24T(shXIAP) cells resulted in a greater invasive ability in comparison to its nonsense transfectant (Fig. S1A, C), while overexpression of Src in T24T(ΔRING) cells attenuated cell invasion in comparison to the scramble vector transfectant (Fig. S1B, D). Our results reveal that Src suppression participates in the XIAP BIR domain’s-mediation of BC cell invasion.

### XIAP BIR domains inhibited Src protein translation through promoting miR-203 expression in human BC cells

Unexpectedly, *Src* mRNA levels were nearly comparable in T24T cells and UMUC3 cells with XIAP knockdown, or XIAP knockdown with either BIR domain overexpression or RING domain overexpression (Fig. S2A). Moreover, depletion of XIAP expression in T24T cells did not result in a slower degradation of Src protein (Fig. S2B), suggesting that XIAP might regulate Src protein translation. The results from incorporation of ^35^S-methionine/cysteine into newly synthesized Src protein in T24T cells in XIAP knockdown cells is markedly increased in comparison to control T24T(nonsense) cells (Fig. S2C), further revealing that XIAP did inhibit Src protein translation. After excluding the possible involvement of S6 ribosomal protein in XIAP inhibition of Src protein translation (Fig. S2D), we tested whether XIAP affected *Src* mRNA 3′-UTR activity. XIAP knockdown resulted in the augmentation of *Src* mRNA 3′-UTR activity, whereas ectopic expression of HA-ΔRING reversed an increase in *Src* mRNA 3′-UTR activity (Fig. S2E), suggesting that the BIR domains are required for XIAP’s inhibition of *Src* mRNA 3′-UTR activity. Since microRNAs (miRNAs) could inhibit protein translation *via* interacting mRNA 3′-UTRs^28^, a bioinformatics analysis was conducted. The results of the bioinformatics analysis show that miR-141, miR-144, miR-137, miR-203, miR-200a, and miR-206 are putative miRNAs that can bind to the 3′-UTR region of *Src* mRNA (Table S1). The results of the evaluation of these putative miRNAs indicate that knockdown of XIAP only attenuated miR-203 expression in T24T cells (Fig. S2F), and the reduction on miR-203 expression was further reversed by ectopic expression of XIAP in the presence of the BIR domains (Fig. S2G). Moreover, the point mutations of the miR-203 binding site in the *Src* mRNA 3′-UTR reporter completely abolished the increased luciferase activity due to XIAP knockdown in T24T cells (Fig. S2H-J). These results reveal that miR-203 directly binds to 3′-UTR of *Src* mRNA and mediates the BIR domains’ inhibition of Src protein translation. Further, overexpression of miR-203 abolished Src protein expression in both T24T(shXIAP) and UMUC3(shXIAP) cells (Fig. S2K-M).

### XIAP BIR domains promoted miR-203 transcription through activation of E2F1 and Sp1

**Fig. 3:**
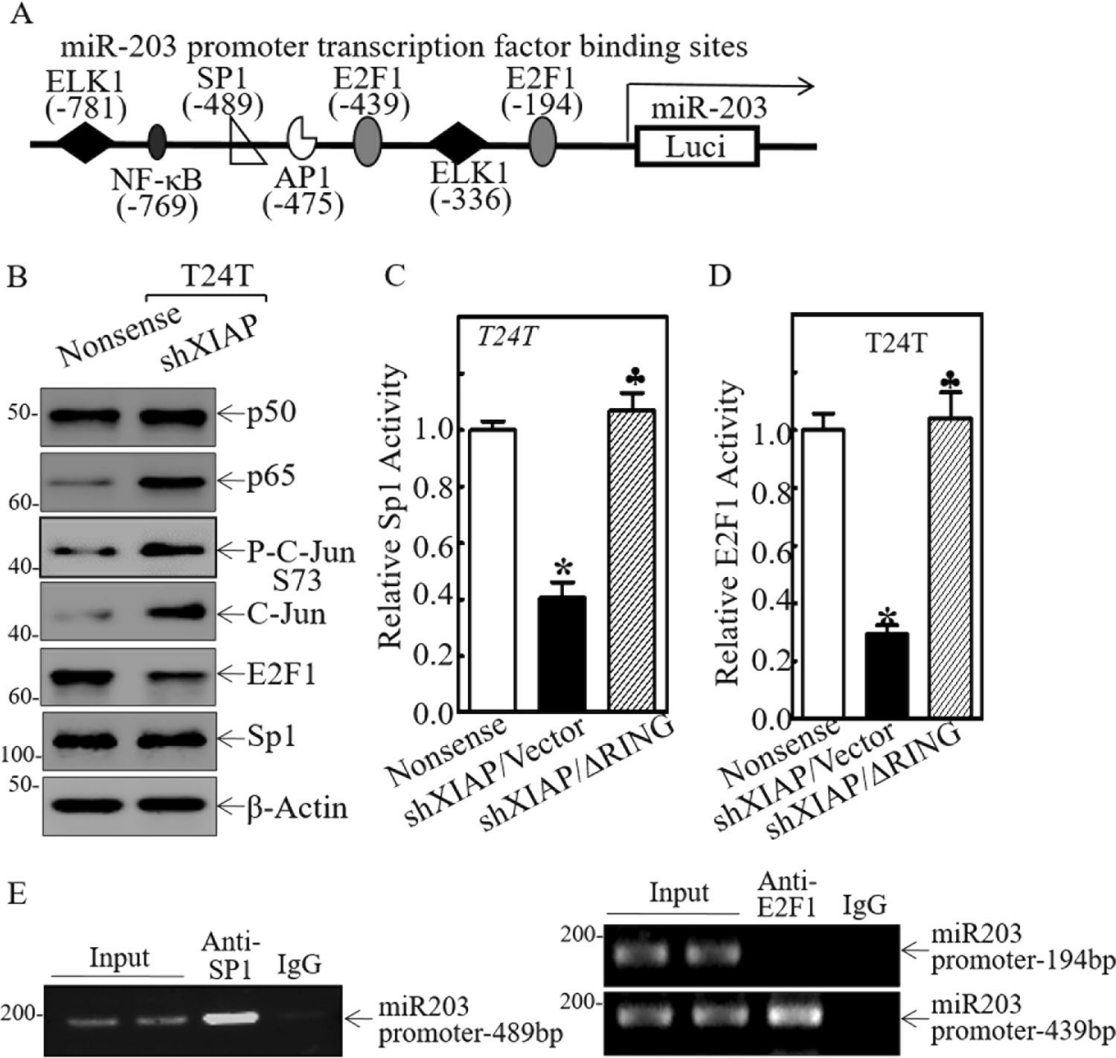
XIAP BIR domains promote transactivation of E2F1 and Sp1 in human BC cells. **a** Schematic representation of the transcription factor binding sites in the human miR-203 promoter-driven luciferase reporter. **b** The indicated cell extracts were subjected to Western blot to determine the functional transcription factors. β-Actin was used as a protein loading control. **c**, **d** T24T (Nonsense), T24T (shXIAP/Vector), and T24T (shXIAP/ΔRING) cells were transfected with a Sp1-dependent luciferase reporter (**c**) or an E2F1-dependent luciferase reporter (**d**), together with pRL-TK. The results are presented as luciferase activity relative to that of the vector control transfectants. **e** ChIP assay was performed using anti-E2F1 or anti-Sp1 antibody to detect the interaction between E2F1 or Sp1 and the miR-203 promoter.

Since miRNAs possess differential stability in human cells^29^, the effect of XIAP/BIR on miR-203 stability was evaluated. Neither inhibition of XIAP expression nor ectopic expression of HA-ΔRING in T24T(shXIAP) cells shows a significant regulatory effect on miR-203 stability as compared with the control transfectants (Fig. S3A). Pre-miRNAs are regulated at transcription and are processed to mature miRNAs by enzymes, such as dicer and argonaute 2^30,31^. To examine whether the BIR domains of XIAP regulate miR-203 at the transcriptional level, we determined the effect of XIAP and its BIR domains on pre-miR-203 expression as well as its promoter activity. The results show that both pre-miR-203 abundance and its promoter-driven luciferase reporter activity are impaired in XIAP knockdown cells, whereas ectopic expression of BIR domains restores both pre-miR-203 expression and its promoter activity (Fig. S3B, C). Given that promoter methylation is an epigenetic mechanism for regulating gene transcription and promoter region demethylation is involved in upregulation of miR-203 as reported in previous studies^32^, we tested whether increase of miR-203 transcription was due to the regulatory effect of BIR domain on demethylation of the miR-203 differentially methylated region (DMR). There was no observable alteration of methylation or unmethylation between T24T cells with either XIAP knockdown cells or BIR domain overexpressed cells, in comparison to parental T24T cells (Fig. S3D). We next bioinformatically analyzed the potential transcription factor binding sites in the miR-203 promoter region. The results reveal that the promoter contains binding sites for multiple transcription factors, including c-Jun, E2F1, Sp1, ELK1, and NFκB (Fig. 3a). The effect of XIAP on these transcription factor expressions was explored in T24T(nonsense) and T24T(shXIAP) cells. The results indicate that knockdown of XIAP in T24T cells only attenuated E2F1 expression, no effect on Sp1 expression and increased p65 and c-Jun (Fig. 3b). However, depletion of XIAP expression in T24T cells dramatically inhibited both E2F1- and Sp1-dependent transcription activity, which could be completely reversed by ectopic expression of ΔRING domain (Fig. 3c, d). These results suggest that inhibition of E2F1- and Sp1-dependent transcription activity might be associated with BIR domain attenuation of miR-203 transcription.

Chromatin immunoprecipitation (ChIP) assays were employed to test the potential direct interaction of Sp1 and E2F1 to their putative binding sites in the miR-203 promoter region. Sp1 did show its binding activity to the miR-203 promoter at −489 bp, whereas E2F1 was only be able to bind at site −439 bp, but not to the putative binding site at −194 bp of the miR-203 promoter region (Fig. 3e). Moreover, overexpression of E2F1 remarkably increased miR-203 expression but did not affect Sp1 expression in both T24T(shXIAP/vector) and UMUC3(shXIAP/vector) cells (Fig. S4A, B), while knockdown of Sp1 not only attenuated miR-203 expression activity in T24T cells (Fig. S4C, D), but also inhibited E2F1 protein expression in T24T cells (Fig. S4C). Consistent with E2F1 and Sp1 promotion of miR-203 transcription, ectopic expression of E2F1 in XIAP knockdown cells increased miR-203 promoter activity (Fig. S4E), while knockdown of Sp1 significantly decreased miR-203 promoter activity (Fig. S4F).

### Sp1 is crucial for BIR domains promotion of E2F1 transcription and BC cell invasion

Interestingly, knockdown of XIAP expression greatly reduced the mRNA level of *E2F1* in both T24T and UMUC3 cells (Fig. S5A). Moreover, depletion of XIAP or Sp1 greatly decreased E2F1 promoter activity, whereas the reduction on E2F1 promoter activity could be reversed completely by ectopic expression of BIR domain (Fig. S5B, C), strongly indicating that the XIAP BIR domains upregulate E2F1 at the transcription level in a Sp1-dependent manner. The Sp1 promotion of E2F1 transcription was also supported by the results from bioinformatics analysis which showed that there are three potential binding sites for Sp1 in the E2F1 promoter region (Fig. S5D). Consistent with the crucial roles that Sp1 and E2F1 play in the modulation of miR-203 transcription, overexpression of E2F1 in T24T(shXIAP) cells markedly increased the cancer cell invasion, while knockdown of Sp1 in T24T cells significantly inhibited the cancer cell invasion (Fig. S5E, F).

### BIR2 and BIR3 specifically interacted with E2F1 and Sp1, respectively, to coordinate the promotion of BC invasion

**Fig. 4:**
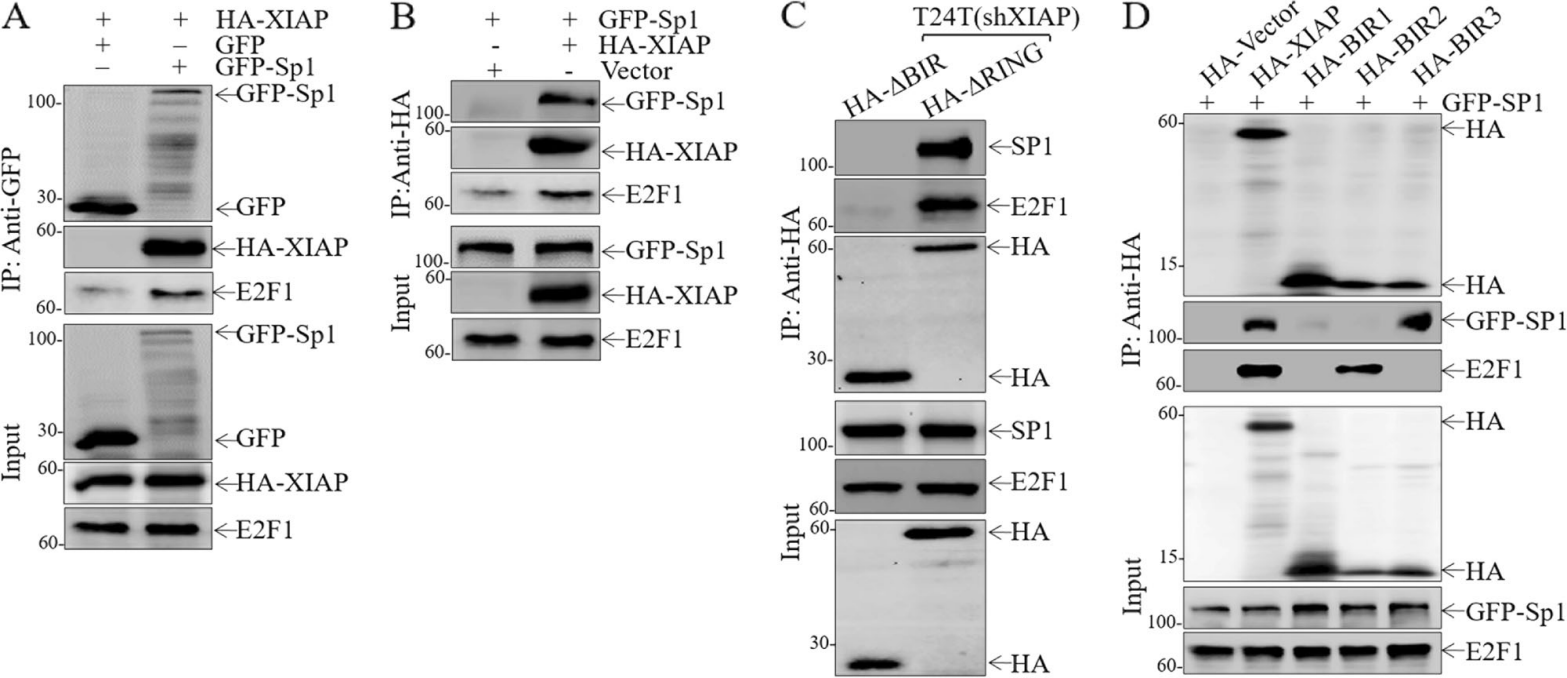
BIR2 and BIR3 domains of XIAP interact differently with Sp1 and E2F1. **a** Immunoblotting analysis of whole-cell lysates (Input) and GFP-immunoprecipitates (IP) obtained from T24T cells transfected with HA-XIAP with or without the combination with GFP-Sp1 using anti-GFP and anti-E2F1 antibody-conjugated beads. **b** Immunoblotting analysis of whole-cell lysates (Input) and HA-immunoprecipitates (IP) obtained from T24T cells transfected with GFP-Sp1 alone or in combination with HA-XIAP using anti-HA and anti-E2F1 antibody-conjugated beads. **c** Total cellular protein was extracted from the indicated cells, and a co-immunoprecipitation assay was performed using anti-HA antibody-conjugated beads. Immunoprecipitated protein was then subjected to Western blotting to detect the interaction of between XIAP BIR domains and antibodies, as indicated. **d** Immunoblotting analysis of whole-cell lysates (Input) and HA-immunoprecipitates (IP) obtained from T24T cells transfected with GFP-Sp1 with or without the combination of various XIAP fragments using anti-HA antibody-conjugated beads.

To elucidate the mechanism of XIAP promotion of Sp1 and E2F1 transcriptional activity, we tested the possibility that XIAP interacts with Sp1. HA-tagged XIAP and E2F1 was present in the immunoprecipitates following anti-GFP antibody pull down of GFP-tagged Sp1 (Fig. 4a). This physical interaction was further demonstrated in the immunoprecipitates using anti-HA antibody to pull down HA-XIAP (Fig. 4b). Even more interesting is that both Sp1 and E2F1 proteins were present in the co-precipitated protein complex in T24T cells in the absence of the XIAP RING domain but not detectable in T24T cells in the absence of the XIAP BIR domains (Fig. 4c), indicating that XIAP interacts with Sp1 and E2F1 through BIR domains in BC cells. Since XIAP contains three BIR domains, we further determined whether Sp1 or E2F1 interacts with XIAP through a specific BIR domain. The results from co-immunoprecipitation assays using anti-HA antibody demonstrate that the BIR2 domain specially interacts with E2F1, while Sp1 specifically bound to the BIR3 domain (Fig. 4d). To further investigate the physiological consequence of this physical interaction between Sp1 and XIAP or E2F1 and XIAP in cells upon serum stimulation, we incubated T24T(HA-XIAP) cells in medium containing 20% FBS for 30 min. The cell extracts were used to perform co-immunoprecipitation assay to pull down endogenous Sp1 and E2F1 using anti-HA antibody. The results show that serum stimulation led to a substantial decrease in XIAP interaction with both Sp1 or E2F1 proteins in BC cells (Fig. S6A). Given that pre-miR-203 transcription occurs in the nucleus, we anticipated that the serum stimulation might result in dissociation of XIAP from E2F1 and Sp1 in BC cells. To test this notion, cytoplasmic and nuclear fractions from T24T cells upon serum stimulation were isolated and further subjected to immunoblotting analysis. Nuclear XIAP translocated to the cytoplasm following 20% FBS stimulation, but E2F1 and Sp1 still stayed in nuclear (Fig. S6B), indicating that nuclear XIAP might be mainly responsible for XIAP interaction with Sp1 and E2F1. Furthermore, ectopic expression of BIR2 and BIR3 show restoration of Src inhibition and MMP2 activation (Fig. S6C) as well as rescued invasion ability (Fig. S6D) in XIAP-deletion BC cells. Consistent with BIR3 promotion of E2F1 transcription via Sp1, only ectopic expression of BIR3, but not BIR2, rescued E2F1 protein expression (Fig. S6C). Given that our published study indicates the inhibition of Rac1 expression by XIAP^10^, it is interesting to define which BIR domain is associated with this function. The results reveal that Rac1 upregulation in XIAP-deficient cells can be specifically abolished by ectopic expression of BIR1, but not BIR2 or BIR3 (Fig. S6C), suggesting that BIR1 mediates XIAP inhibition of Rac1 expression. Consistent with the activation of MMP2 by BIR2 and BIR3, ectopic expression of BIR2 and BIR3 also restored E2F1- and Sp1-dependent transactivation (Fig. S7A, B), miR-203 promoter activation, and miR-203 expression (Fig. S7C, D).

## Discussion

Our current study shows that the BIR domains of XIAP is one of the major factors in the promotion of human BC cell invasion. This important function of the XIAP/BIR domains is mediated *via* specific activation of MMP2. Matrix metalloproteinases-2 (MMP2) belongs to one of the gelatinases that are primary subgroups of MMPs on the premise of domain structure^14^. MMP2 has a well-known role in degradation of connective tissue stroma and basement membranes and is a good candidate to be a biological marker in many cancers^33^. MMP2 is secreted into the matrix as pro-MMP2 with an auto-inhibitory N-terminal pro-domain^34^. The cysteine switch motif in this domain blocks the catalytic zinc, preventing hydrolysis of substrates^35^. Pro-MMP2 can be activated *via* proteolytic cleavage or chemical disruption of the pro-domain to expose the catalytic zinc to enzyme activity^15^. MMP2 activation has been reported to correlate directly with the level of aggressiveness in bladder tumors^36^. Here, we are the first to unravel a novel function for XIAP in the promotion of MMP2 activation that is attenuated by the increased translation level of proto-oncogene tyrosine-protein kinase Src. We establish a new bridge between XIAP overexpression and MMP2 activation in human BC high invasion, and further help us better understand XIAP induction associated with the progression and the aggression of malignant bladder tumor development^37^. Further investigation will mainly focus on the precise role of XIAP in vivo using XIAP knockout, or overexpression, as well as each BIR domain deletion knock-in mouse model.

The function of Src in cancer biology, in general, is dependent on the cancer types. For example, Src is overexpressed or activated in breast, prostate, colorectal, pancreatic, hepatocellular, esophageal, head and neck, ovarian, and lung cancer, as well as in leukemia, and lymphoma^38^. Src is the oldest and best-studied proto-oncogene, and its high expression or activation is positively associated with tumor grade and stage in these cancers^39,40^. However, Src protein levels are attenuated with increases in the levels of BC stages as well as areas that are affected by metastasis^27,41^. Our current study revealed low mRNA and protein expression of Src in human and mouse BC tissues. Thus, Src is a potential tumor suppressor in BCs. To the best of our knowledge, our discovery that Src-mediated BC invasion mainly relies on its downstream powerful BC invasion/metastatic effector, MMP2 is without precedent. We have also demonstrated that Src-associated MMP2 regulation only targets MMP2 activation rather than its expression in BIR domains of XIAP-dependent. These new findings not only help us be more aware of the tumor suppressive role of Src in BCs, but also warns us to consider the tissue-specificity of drugs targeting Src. Further study needs to be elucidated to determine how Src-regulates MMP2 activation.

It has previously been reported that miR-203 is increased in bladder cancers^42^, indicating that it may function as a tumor promoter in the disease’s progression. In the present studies, we find that miR-203 has an essential role in XIAP regulation of Src expression *via* binding to 3′-UTR of *Src* mRNA. Consistent with its oncogenic role of XIAP in Src protein expression, we found that miR-203 significantly inhibits Src protein translation without affecting its mRNA. Furthermore, we have shown that XIAP promotes miR-203 expression through enhancing Sp1 and E2F1 activation. Sp1 and E2F1 are both transcription factors, and their expression and activity has been reported to be elevated in many cancers^43–45^. Here, we report that XIAP might act as a promising natural promoter of Sp1 and E2F1 through specific interaction with them that enhances their activity. Moreover, we found that Sp1 or E2F1 are mainly bound to nuclear XIAP in unstimulated BC cells, but dissociated with XIAP when nuclear XIAP shuttled to cytoplasm following serum stimulation. Additionally, the molecular mechanism that mediates the dissociation of Sp1 and E2F1 from XIAP in BC cells upon serum stimulation also merits further investigation. Our previous report reveals that in HCT116 colon cancer cells, the BIR domains of XIAP could bind E2F1 to promote cell growth by strengthening cyclin E expression^9^. Our current finding further extends this knowledge, revealing that E2F1 binds to the XIAP BIR2 domain as well as the discovery that Sp1 interacts with the XIAP BIR3 domain, and that Sp1 also acts as an E2F1 upstream transcriptional factor to initiate a crosstalk with E2F1 to promote BC cell invasion. The crosstalk between the BIR2 and BIR3 domains in the regulation of miR-203/SRC/MMP2 axis is greatly supported by the findings that ectopic expression of either BIR2 or BIR3 could restore E2F1-dependent transactivity, whereas only BIR3, but not BIR2, rescued Sp1-dependent transactivity.

In summary, our studies reveal a novel Sp1/E2F1/miR-203/Src pathway that is responsible for the activation of MMP2 and the tumor-promotive role of XIAP in BC cell invasion. We show a new link between XIAP, Src, and MMP2 activation, which may be BC specific. Moreover, we identify two physical protein-protein interactions: XIAP and Sp1, XIAP and E2F1, and further point out that the BIR2 domain of XIAP is essential and sufficient for its interaction with E2F1, while BIR3 domain of XIAP is primarily responsible for its binding with Sp1. In addition, we find that BIR3 of XIAP-initiated Sp1 also acts as an upstream regulator for E2F1 transcription. Although the detailed mechanisms underlying this observation have not yet been completely discovered, the comprehensive feedback regulatory pathways might be involved. Further elucidation of this issue will be helpful for understanding XIAP domain-based function in regulation of BC invasion.

## Supplementary information

Supplement of Materials and Methods

Supplementary Legends

Supplementary Table

Supplementary Figure 1

Supplementary Figure 2

Supplementary Figure 3

Supplementary Figure 4

Supplementary Figure 5

Supplementary Figure 6

Supplementary Figure 7
