# Supplementary material for: XIAP Interaction with E2F1 and Sp1 via its BIR2 and BIR3 domains specific activated MMP2 to promote bladder cancer invasion
Source: Oncogenesis. 2019 Dec 6;8(12):71. doi: 10.1038/s41389-019-0181-8 (PMC6898186; doi:10.1038/s41389-019-0181-8)
Supplement: Supplementary file 1 — Supplement of Materials and Methods [file 41389_2019_181_MOESM1_ESM.docx]

**Supplement of Materials and Methods**

**Cell lines, plasmids, antibodies, and other reagents**

UMUC3 cells were maintained in Dulbecco’s modified Eagle’s medium (DMEM) supplemented with 10% FBS (HyClone, Logan, UT), 1% penicillin/streptomycin and 2 mM L-glutamine (Life Technologies, Rockville, MD). T24T cells were cultured in DMEM/Ham's F-12 (1:1 volume) mixed medium supplemented with 5% FBS, 1% penicillin/streptomycin and 2 mM L-glutamine.

The shRNA that specifically targets human XIAP and Sp1 was purchased from Open Biosystems (GE, Pittsburgh, PA). HA-∆BIR and HA-∆RING expression plasmids were described in our previous studies ([1-3](#_ENREF_1)). miR-203 mimic RNA was kindly provided by Dr. Dale D. Tang (The Center for Cardiovascular Sciences, Albany Medical College, Albany, New York) ([4](#_ENREF_4)). The Src expression plasmid was obtained from Addgene (Cambridge, MA). E2F1- and Sp1-dependent luciferase reporters were described in our previous papers ([1](#_ENREF_1), [5](#_ENREF_5)). The human Src mRNA 3’-UTR luciferase reporter and its mutant (the binding site of miR-203 was mutated) was cloned into a pMIR-report luciferase vector. The plasmid containing the luciferase reporter under the control of human miR-203 gene promoter was constructed into a PGL3-Basic vector.

Anti-XIAP antibody (BDB610763) was purchased from Becton, Dickinson and Company (Franklin Lakes, NJ). Specific antibodies against HA (# 3724S), Src (# 2109S), S6 ribosomal protein (# 2217S), P-S6 ribosomal protein Ser235/236 (# 4858S), p53 (# 9282S), c-Jun (# 9165S), P-c-Jun at Ser73 (# 3270S), NF-κB p65 (# 8242S), and GAPDH (# 5174S) were purchased from Cell Signaling Technology (Beverly, MA). Antibodies specific for Sp1 (sc-14027), E2F1 (sc-193), MMP2 (sc-6838), and β-Actin (sc-47778), were bought from Santa Cruz (Dallas, TX). Antibodies specific against p50 (ab32360) were bought from Abcam (Cambridge, MA, USA).

The protein synthesis inhibitor cycloheximide (CHX) was purchased from Calbiochem (San Diego, CA, USA). The dual luciferase assay kit was purchased from Promega (Madison, WI, USA). TRIzol reagent and the SuperScript™ First-Strand Synthesis system were bought from Invitrogen (Grand Island, NY, USA). PolyJet™ DNA In-Vitro Transfection Reagent was purchased from SignaGen Laboratories (Rockville, MD, USA). Both the miRNeasy Mini Kit and the miScript PCR system for miRNA detection were bought from Qiagen (Valencia, CA, USA).

**Human bladder cancer tissue samples**

All specimens were immediately snap-frozen in liquid nitrogen after surgical removal. Histological and pathological diagnoses were confirmed, and the specimens were classified by a certified clinical pathologist according to the standards of the 2004 World Health Organization Consensus Classification and Staging System for bladder neoplasms. All specimens were obtained with appropriate informed consent from the patients. A supporting grant was obtained from the Medical Ethics Committee of China. The experiments were carried out in accordance with The Code of Ethics of the World Medical Association (Declaration of Helsinki) for experiments involving human studies.

**Animal experiments and immunohistochemistry-paraffin (IHC-P)**

Male C57BL/6J mice, 5~6 weeks old, were randomly divided into two groups of 12, including a vehicle-treated control group and an N-butyl-N-(4-hydroxybutyl) nitrosamine (BBN)-treated group. Mice in the BBN-treated group received BBN (0.05%) in drinking water for 20 weeks, while the vehicle-treated group was provided with normal drinking water containing same amount of DMSO. The mice were euthanized at the end of the experiment. Mouse bladder tissues were excised and fixed overnight in 4% paraformaldehyde at 4°C. Fixed tissues were processed for paraffin embedding, and the serial 5-μm-thick sections were then immunostained with specific antibodies against Src (Cell Signaling Technology, MA USA). The resultant immunostaining images were captured using an AxioVision Rel.4.6 computerized image analysis system (Carl Zeiss, Oberkochen, Germany). Protein expression levels were examined by the integrated optical density per stained area (IOD/area) that was analyzed with Image-Pro Plus, version 6.0 (Media Cybernetics, MD). Briefly, the IHC stained sections were evaluated at 400-fold magnification, and at least 5 representative staining fields in each section were analyzed to calculate the optical density based on typical images that had been captured.

**Western blot**

Briefly, cells were plated in 6-well plates and cultured in normal FBS medium until 70–80% confluent. The cells were then cultured in 0.1% FBS medium for 12 hours, followed by treatment with different doses of ISO for the timer periods indicated. The cells were washed once with ice-cold phosphate-buffered saline, and cell lysates were prepared with a lysis buffer (10 mM Tris-HCl (pH 7.4), 1% SDS, and 1 mM Na3VO4). An equal amount (80 μg) of total protein from each cell lysate was subjected to Western blot with the indicated antibody. Immunoreactive bands were detected using alkaline phosphatase-linked secondary antibody and an ECF Western blotting system (Amersham Biosciences, Piscataway, NJ). Images were acquired using a Typhoon FLA 7000 imager (GE Healthcare, Pittsburgh, PA).

**RT–PCR and quantitative RT-PCR**

A pair of oligonucleotides (Forward: 5’- GAT GAT CTT GAG GCT GTT GTC -3’ and Reverse: 5’- CAG GGC TGC TTT TAA CTC TG -3’) were used to amplify human GAPDH cDNA as a loading control. The human Src cDNA fragments were amplified with a pair of human Src-specific PCR primers (Forward: 5’-TCC GAC TCC ATC CAG GCT GA -3’ and Reverse: 5’- TGT CCA GCT TGC GGA TCT TG -3’). The human E2F1 cDNA fragments were amplified with 5’- GAG GTG CTG AAG GTG CAG AA-3’; (Forward) and 5’- GTT TGC TCT TAA GGG AGA TCT G -3’ (Reverse). The PCR products were separated on 2% agarose gels, stained with ethidium bromide (Fisher Scientific Corporation, MA, USA), and scanned for imaging under UV light. The results were visualized with a Αlpha Innotech SP Imaging System (Αlpha Innotech Corporation, San Leandro, CA, USA). Quantitative RT-PCR was performed to examine the expression level of mature miRNAs and pre-miRNA, as described previously ([6](#_ENREF_6)).

**[^35^S] Methionine pulse new protein synthesis assays**

Cells were incubated with methionine-cysteine free DMEM (Gibco-BRL, Grand Island, NY, USA) containing 2% dialyzed fetal calf serum (Gibco-BRL) and 10 μM MG132 for 30 minutes, then incubated with 2% FBS methionine-cysteine-free DMEM containing ^35^S-labeled methionine/cysteine (250 μCi per dish, Biomedicals, Inc., Irvine, CA) for the indicated periods. The cells were extracted with lysis buffer (Cell Signaling Technology, MA) containing a complete protein inhibitor mixture (Roche, Swiss) on ice, and 500 mg of total lysate was incubated with anti-Src antibody-conjugated agarose beads (R&D Systems, Minneapolis, MN, USA) overnight at 4°C. The immunoprecipitates were washed five times with the cell lysis buffer, heated at 100°C for 5 min, then subjected to sodium dodecyl sulfate polyacrylamide gel electrophoresis. The membranes were then subjected to autoradiography to determine the newly synthesized ^35^S-labeled Src protein, as described in our previous studies ([5](#_ENREF_5), [7](#_ENREF_7)).

**Luciferase assay**

T24T and UMUC3 cells were transfected with the indicated luciferase reporter constructs in combination with a pRL-TK vector (Promega, Madison, WI). The transfectants were seeded into 96-well plates and cultured for 12 hours. The cells were then extracted with 1x Passive Lysis Buffer (Promega, Madison, WI) and subjected to determine luciferase activity using a luciferase assay system (Promega Corp., Madison, WI) with a microplate luminometer LB 96V (Berthold GmbH & Co. KG, Bad Wildbad, Germany). The luciferase activity was normalized to the internal control TK activity based on the manufacturer’s instructions.

**Methylation-speciﬁc PCR**

Genomic DNA was isolated with a DNeasy Blood & Tissue Kit (Qiagen, Hilden, Germany) according to the manufacturer's instructions. Genomic DNA (2 µg) was treated with sodium bisulﬁte using an EpiTect Bisulﬁte Kit (Qiagen, Hilden, Germany). Methylation-speciﬁc PCR was performed using 20 ng of bisulﬁte-converted DNA and speciﬁc primers. Methylated primers and unmethylated primers for the miR-203 promoter at the differentially methylated region (DMR) were designed according to a previous study ([8](#_ENREF_8)). PCR products were run on a 2% agarose gel and visualized after ethidium bromide staining. Bisulﬁte-converted methylated and unmethylated DNA from the EpiTect PCR Control DNA Set (Qiagen, Hilden, Germany) were used as positive and negative controls.

**Immunoprecipitation**

For immunoprecipitation experiments, cells transfected with the indicated plasmids were collected and lysed in 1 × Cell Lysis Buffer (Cell Signaling Technology, Danvers, MA, USA) containing protease inhibitors (Roche, Branchburg, NJ, USA) followed by brief sonication. Any insoluble material was removed by centrifugation at 16,000×g for 20 minutes at 4°C. Immunoprecipitation was carried out by incubation of cell lysates with anti-HA or anti-GFP antibody-conjugated agarose beads. After overnight incubation, beads were washed three times with immunoprecipitation lysis buffer and bound proteins were subjected to Western blot assay ([6](#_ENREF_6)).

***In vitro* cell migration and invasion assays**

In vitro migration and invasion assays were conducted using transwell chambers (for migration assays) or transwell chambers pre-coated with Matrigel (for invasion assays), according to the manufacturer's protocol (BD Biosciences, Bedford, MA), as previously described ([9](#_ENREF_9)). Briefly, 700 μl of medium containing FBS (10% for UMUC3 and 5% for T24T cells with different transfectants) was added to the lower chambers, while homogeneous single cell suspensions (5×10^4^ cells/well) in 0.1% FBS medium was added to the upper chambers. The transwell plates were incubated in a 5% CO_2_ incubator at 37℃ for 24 hours and thereafter washed with PBS, fixed with 4% formaldehyde, and stained with Giemsa stain. The non-migrating or non-invading cells were scraped off from the top of the chamber. The migration and invasion rates were quantified by counting the migratory and invasive cells in at least three random fields under a light microscope (Olympus, Center Valley, PA).

**Statistical methods**

Associations between the categorical variables were assessed using a chi-square test. Student’s t-test was utilized to compare continuous variables, and the results are summarized as the mean ± SD between different groups. Paired t-tests were performed to compare the difference between paired tissues in the real-time PCR analysis. p < 0.05 was considered statistically significant.

**References**

1. Jin H, Xu J, Guo X, Huang H, Li J, et al. XIAP RING domain mediates miR-4295 expression and subsequently inhibiting p63alpha protein translation and promoting transformation of bladder epithelial cells. Oncotarget. 2016.

2. Cao ZP, Li XY, Li JX, Luo WJ, Huang CS, et al. X-linked inhibitor of apoptosis protein (XIAP) lacking RING domain localizes to the nuclear and promotes cancer cell anchorage-independent growth by targeting the E2F1/Cyclin E axis. Oncotarget. 2014;5(16):7126-37.

3. Cao ZP, Zhang RW, Li JX, Huang HS, Zhang DY, et al. X-linked Inhibitor of Apoptosis Protein (XIAP) Regulation of Cyclin D1 Protein Expression and Cancer Cell Anchorage-independent Growth via Its E3 Ligase-mediated Protein Phosphatase 2A/c-Jun Axis. J Biol Chem. 2013;288(28):20238-47.

4. Liao G, Panettieri RA, Tang DD. MicroRNA-203 negatively regulates c-Abl, ERK1/2 phosphorylation, and proliferation in smooth muscle cells. Physiol Rep. 2015;3(9).

5. Yu Y, Zhang D, Huang H, Li J, Zhang M, et al. NF-kappa B1 p50 promotes p53 protein translation through miR-190 downregulation of PHLPP1. Oncogene. 2014;33(8):996-1005.

6. Wang Y, Xu J, Gao G, Li J, Huang H, et al. Tumor-suppressor NF kappa B2 p100 interacts with ERK2 and stabilizes PTEN mRNA via inhibition of miR-494. Oncogene. 2016;35(31):4080-90.

7. Hua X, Huang M, Deng X, Xu J, Luo Y, et al. The inhibitory effect of compound ChlA-F on human bladder cancer cell invasion can be attributed to its blockage of SOX2 protein. Cell Death Differ. 2019.

8. Noguchi S, Mori T, Nakagawa T, Itamoto K, Haraguchi T, et al. DNA methylation contributes toward silencing of antioncogenic microRNA-203 in human and canine melanoma cells. Melanoma Res. 2015;25(5):390-8.

9. Jiang G, Wu AD, Huang C, Gu J, Zhang L, et al. Isorhapontigenin (ISO) Inhibits Invasive Bladder Cancer Formation In Vivo and Human Bladder Cancer Invasion In Vitro by Targeting STAT1/FOXO1 Axis. Cancer Prev Res (Phila). 2016;9(7):567-80.
