# Supplementary material for: XIAP Interaction with E2F1 and Sp1 via its BIR2 and BIR3 domains specific activated MMP2 to promote bladder cancer invasion
Source: Oncogenesis. 2019 Dec 6;8(12):71. doi: 10.1038/s41389-019-0181-8 (PMC6898186; doi:10.1038/s41389-019-0181-8)
Supplement: Supplementary file 2 — Supplementary Legends [file 41389_2019_181_MOESM2_ESM.docx]

**Supplementary Figure Legends**

**Fig. S1** **Src downregulation by XIAP BIR domains result in BC invasion in human BCs. (A and B)** The cell extracts from the indicated stable transfectants were subjected to Western Bot to determine related protein expression. **(C-D)** The indicated stable transfectants were subjected to cell migration and invasion assays as described in “Materials and methods”. Original magnification, ×100. Scale bars, 10 µm. All data represent the mean ± SD of 3 independent experiments and a p < 0.05 (*) was statistically significant.

**Fig. S2 BIR domains of XIAP promoted miR-203 transcription and in turn, inhibited Src tyrosine kinase protein translation in human bladder cancer cells. (A)** Total RNA was isolated from the indicated cells, then subjected to RT-PCR analysis of src mRNA expression. GAPDH was used as a loading control. **(B)** After pre-treatment with MG132 (10 μM) for 6 h, T24T (Nonsense) and T24T (shXIAP) cells were subjected to determine Src protein degradation in the presence of cycloheximide (CHX) (100 μg/ml). β-Actin was used as a protein loading control. **(C)** After pretreatment with MG132 (10 μM) for 30 minutes, newly synthesized Src protein in T24T (Nonsense) and T24T (shXIAP) cells was monitored with a pulse assay using ^35^S-labeled methionine/cysteine. WCL stands for whole-cell lysate. Coomassie blue staining was used as the protein loading control. **(D)** The cell extracts were subjected to Western Blot, as indicated. β-Actin was used as the loading control. **(E)** The indicated cells were transiently transfected with a Src 3′UTR luciferase reporter, and the luciferase activity of each transfectant was evaluated. The results are presented as Src 3′-UTR activity relative to the medium control. The symbol (*) indicates a significant increase compared with T24T (Nonsense/Vector) (p < 0.01). The symbol (^♣^) indicates a significant decrease compared with T24T (shXIAP/Vector) cells. **(F)** The levels of the indicated microRNAs were evaluated with quantitative real-time PCR. The symbol (*) indicates a significant decrease compared with control cells, as indicated (p < 0.01). **(G)** The level of miR-203 in the indicated cells was evaluated with quantitative real-time PCR. The symbol (*) indicates a significant decrease compared with the nonsense cells, as indicated (p < 0.01), while the symbol (^♣^) indicates a significant increase as compared with T24T (shXIAP/Vector) cells. **(H)** Schematic of the construction of the src mRNA 3′-UTR luciferase reporter and its mutants aligned with miR-203. **(I and J)** T24T (Nonsense), T24T (shXIAP) cells, and T24T (shXIAP/Vector), T24T (shXIAP/ΔRING) cells were co-transfected with wild-type and mutant src 3′-UTR luciferase reporters and pRL-TK, respectively. The luciferase activity of each transfectant was evaluated and the results are presented as relative to src 3′-UTR activity. The symbol (*) indicates a significant difference in src 3′-UTR activity (p < 0.01). **(K)** T24T (shXIAP/Vector) and UMUC3 (shXIAP/Vector) cells were stably transfected with constructs of miR-203 or its control vector. miR-203 expression was determined with real-time PCR, and the symbol (*) indicates a significant increase compared with the control nonsense transfectant (p < 0.05). **(L and M)** The indicated cell extracts were subjected to Western blotting, and β-Actin was used as the protein loading control. All data represent the mean ± SD of 3 independent experiments and (*) or (^♣^) was statistical significant (p < 0.05).

**Fig. S3 XIAP BIR domains promote miR-203 transcription. (A)** The indicated cells were incubated with actinomycin D (20 μg/ml) for the indicated time periods. Total RNA was isolated and quantitative real-time PCR was then performed to determine miR-203 levels. The fold change was normalized using GAPDH as the internal control. **(B)** The relative expression levels of pre-miR203 were evaluated with quantitative real-time PCR in the indicated cells. **(C)** The indicated cells were stably transfected with a miR-203 promoter-driven luciferase reporter to determine the miR-203 promoter transcriptional activity. **(D)** XIAP and its BIR domains did not affect miR-203 promoter methylation. All data represent the mean ± SD of 3 independent experiments and (*) or (^♣^) was statistical significant (p < 0.05).

**Fig. S4 XIAP BIR domains promote miR-203 transcription through transactivation of E2F1 and Sp1 in human BC cells. (A and B)** T24T and UMUC3 cells stably transfected with E2F1 overexpression construct, and the stable transfectants were identified and Sp1 expression was determined (A). (B) The stable transfectants were subjected to evaluate the level of miR-203 with real-time PCR. The symbol (*) indicates a significant increase in miR-203 expression in E2F1 overexpression cells compared with the vector transfectants (p < 0.01). **(C and D)** T24T cells were stably transfected with two Sp1 knockdown plasmids separately and Western blot was employed to determine Sp1 protein expression. Real-time PCR was performed to determine the miR-203 expression in the stable Sp1 knockdown cells. **(E)** The stable E2F1 overexpressed BC cells were stably transfected with a miR-203 promoter-driven luciferase reporter to determine the miR-203 promoter transcriptional activity. **(F)** The stable Sp1 knockdown cells were stably transfected with a miR-203 promoter-driven luciferase reporter to determine the miR-203 promoter transcriptional activity. All data represent a mean ± SD of 3 independent experiments and (*) or (^♣^) was statistically significant (p < 0.05).

**Fig. S5 Sp1 is crucial for XIAP BIR domains’-mediated E2F1 transcription and promotion of BC cell invasion.** **(A)** RT-PCR was performed to determine the e2f1 mRNA levels in the indicated cells. **(B)** T24T (Nonsense), T24T (shXIAP/Vector), and T24T (shXIAP/ΔRING) cells were co-transfected with an E2F1 promoter-driven luciferase reporter together with pRL-TK. 24 hours post transfection, the transfectants were extracted to evaluate luciferase activity, with normalization to TK. The results are presented as luciferase activity relative to scramble nonsense transfectant. Each bar indicates the mean ± SD of three independent experiments. The symbol (*) and (♣) indicates a significant difference compared with the vehicle control and T24T (shXIAP/vector), separately (p < 0.05). **(C)** The stable Sp1 knockdown cells were stably transfected with E2F1 promoter-driven luciferase reporter to determine the E2F1 promoter transcriptional activity. **(D)** The potential transcription factor binding sites in the e2f1 promoter. **(E-F)** Different types of transfectants were subjected to cell invasion and migration assays with transwell invasion assay system. Original magnification, ×100. Scale bars, 10 µm. The migration and invasion rates were normalized with the insert control according to the manufacturer’s instructions. The results are presented as the number of migratory or invasive cells relative to vector control transfectants. All data represent a mean ± SD of 3 independent experiments and (*) or (^♣^) was statistical significant (p < 0.05).

**Fig. S6 BIR2 and BIR3 domains of XIAP promote BC invasion.** **(A)** Immunoblotting analysis of whole-cell lysates and anti-HA-immunoprecipitates (IP) obtained from T24T(HA-XAP) cells following synchronization overnight in 0.1% fetal bovine serum (FBS) medium and further stimulation with 20% FBS medium for 30 min. **(B)** Immunoblotting analysis of cytoplasmic and nuclear fractions of T24T cells following 24 h of serum deprivation and further stimulation with 20% FBS for 30 min. Two XIAP binds come from the same WB membrane: upper: long exposure, lower: short exposure. β-Actin and poly-(ADP-ribose) polymerase (PARP) are cytoplasmic and nuclear markers, respectively. **(C)** The indicated cell extracts were subjected to Western blotting to determine the expression of XIAP, E2F1, Src, pro-MMP2, and cleaved-MMP2 (activated-MMP2). β-Actin was used as the protein loading control. **(D)** Different types of transfectants were subjected to cell invasion and migration assay using the transwell invasion assay system. Original magnification, ×100. Scale bars, 10 µm. The migration and invasion rates were normalized with the insert control according to the manufacturer’s instructions, and the symbol (*) and (♣) indicates a significant difference compared to the vehicle control and T24T (shXIAP/vector), separately (p < 0.05).

**Fig. S7 XIAP BIR domains mediated E2F1 and Sp1 activity. (A and B)** T24T (Nonsense), T24T (shXIAP/Vector), T24T (shXIAP/BIR1), T24T (shXIAP/BIR2), and T24T (shXIAP/BIR3) cells were transfected with an E2F1-dependent luciferase reporter (A) or a Sp1-dependent luciferase reporter (B), together with pRL-TK. The results are presented as luciferase activity relative to that of the vector control transfectants. **(C)** The indicated cells were stably transfected with a miR-203 promoter-driven luciferase reporter to determine the miR-203 promoter transcriptional activity. The symbol (*) indicates a significant decrease compared to the nonsense cells, as indicated (p < 0.01), while the symbol (♣) indicates a significant increase compared to the T24T (shXIAP/Vector) cells. **(D)** The level of miR-203 in the indicated cells was evaluated with quantitative real-time PCR. **(E)** The schematic of the potential XIAP BIR domains- and RING domain-mediated regulation of bladder cancer promotion and invasion. All data represent a mean ± SD of 3 independent experiments and (*) or (^♣^) was statistical significant (p < 0.05)
