# Supplementary figures and images for: XIAP Interaction with E2F1 and Sp1 via its BIR2 and BIR3 domains specific activated MMP2 to promote bladder cancer invasion
Source: Oncogenesis. 2019 Dec 6;8(12):71. doi: 10.1038/s41389-019-0181-8 (PMC6898186; doi:10.1038/s41389-019-0181-8)

Table S1: The potential miRNAs binding sites in the Src mRNA 3’UTR region


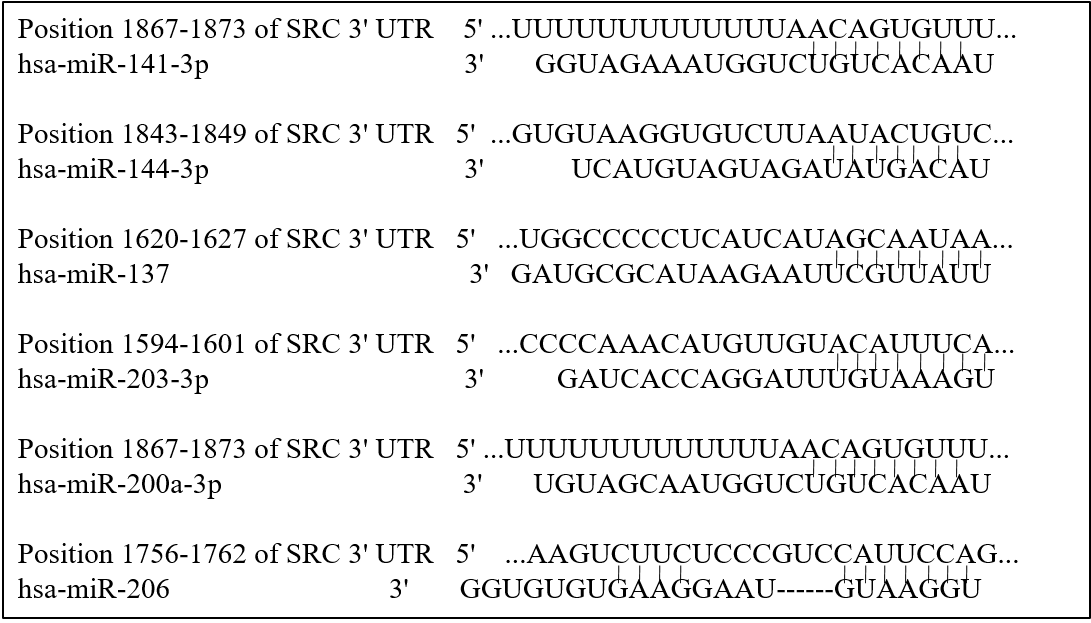

Supplement: Supplementary file 3 — Supplementary Table [file 41389_2019_181_MOESM3_ESM.docx]
